# Self-motivated effects of teachers’ supportive behaviors on students’ intentions of online continuous learning -- based on educational digital transformation

**DOI:** 10.1371/journal.pone.0324731

**Published:** 2025-06-05

**Authors:** Zhiqun Ouyang, Xin Bai, Lin Huang, Qiang Li, Yingji Li

**Affiliations:** 1 Hunan International Economics University, Changsha, China; 2 College of Architectural Arts, Guangxi Arts University, Nanning, China; 3 Stamford International University, Bangkok, Thailand; 4 Shanghai Technical Institute of Electronics & Information, Shanghai, China; 5 Yunnan University of Chinese Medicine, Kunming, China; University of Central Punjab, PAKISTAN

## Abstract

The study explored the factors influencing students’ online continuous learning intentions (CLI) based on educational digital transformation (EDT). The theoretical model was based on the technology acceptance model (TAM), and two dimensions were proposed: flow experience (FE) and teachers’ supportive behaviors (TSBs). It studied how these variables directly or indirectly affected students’ intentions to CLI within EDT. The constructed model was validated by exploiting a partial least squares structural equation modeling approach (PLS-SEM) based on the valid data from 614 students from five private universities in China. The results suggested that (a) the construct TSBs, including teachers’ social support, teachers’ intellectual support, and teachers’ instrument support, had a positive impact on students’ intentions of online continuing learning within EDT; (b) perceived ease of use (PEU) had a positive effect on perceived usefulness (PU), flow experience (FE) and CLI; (c) perceived usefulness (PU) had an effect on FE and CLI; (d) flow experience (FE) had a positive effect on CLI. The findings offered valuable insights for academicians, higher institution administrators, researchers, and higher education policy-makers in enhancing students’ learning based on educational digital transformation.

## 1. Introduction

The phenomenon of high dropout rates and low completion rates of online learning has always been a concern to educators and researchers, hindering the development of online courses [1–3]. Students requires stronger intentions to peisist in online learning enviroments. The intention to continue learning encompasses many aspects, such as learners’ cognition, behavior, motivation, and emotional regulation, reflecting psychological commitment to overcome distractions, learning barriers, and environmental uncertainties during online education processes [4]. Current research‌ generally conceptualizes ‌sustained learning intention‌ at two distinct levels: ‌learners’ intention to complete specific ‌learning objectives‌ within the current course and ‌their‌ intention to pursue subsequent ‌educational programs‌ in the same domain [5]. This study specifically examines‌ the behavioral intention ‌regarding‌ current course completion, ‌with particular focus on‌ self-regulated learning strategies and ‌institutional support mechanisms.

Existing research examines the influencing factors of continuous learning intention from multiple dimensions [6]. In terms of learners’ individual characteristics, motivation serves as a primary determinant of continuous learning intention [7]. Additionally, academic background, learning capabilities, and prior learning experiences significantly influence the intention of continuing learning [8]. Regarding the learning environment, key factors include task design characteristics, immersion level, interaction quality, sense of presence, and learning support [9]. From the perspective of perceived experience, students’ self-efficacy, teaching presence, perceived usefulness, and perceived ease of use are identified as critical predicators of continuous learning intention. Theoretical models posit that‌ enhancing learning satisfaction ‌may serve as a catalyst for strengthening‌ continuous learning intention [10].

Current studies have systematically analyzed factors influencing continuous online learning intentions through multiple lenses. From the course designed perspective, curriculum relevance, utility, effectiveness, teacher-student interaction dynamics, instructors’ personal attributes, and pedagogical strategies are identified as critical determinants [9]. Technical and task-related factors**‌** including essential task characteristics, technological compatibility, task-technology alignment, and user satisfaction exert significant impacts on online learning persistence [11]. Instructional interaction mechanisms**‌** encompassing interaction design principles, learning support infrastructure, motivational drivers, interaction proximity, and balanced autonomy-collaboration activities further predict continuance intentions [12]. Students’ psychology constructs such as academic self-efficacy, teaching presence, perception of usefulness, perception of usefulness, and cognitive engagement emerge as decisive predictors [13,14]. From the learning environment perspective, Baber believes that interaction modalities, such as interactions of human-machine, peer-to-peer, and human-content, immersive experiences, and satisfaction, will also affect the willingness to continue learning online [15]. Previous knowledge, internal interest, and self-identity also affect continuous online learning [16]. Wang claims that it is expected to confirm, perceive the usefulness, perceive entertainment, perceived ability, perceived autonomy, perceived relationship, internal motivation, self-efficacy, and factors that affect online learning willingness [17]. From the perspective of self-efficacy, teaching sense, perception of usefulness, perception of ease of use, and learning input, Aldhahi finds factors affecting continuous online learning [18]. Shahzad believes that objective quality, perception quality, perception of usefulness, perceived value, motivation incentives, learner satisfaction, and subjective norms have greatly affected the willingness to continue learning online [19]. Rajeh identified factors affecting online continuous learning from psychological levels, such as perception scarcity, lack of control, psychological resistance, and focus on video lectures [20]. Some scholars summarize from attribution theory. Ferrer believes that successful and failure attribution, internal motivation, and external motivation will affect online continuous learning intentions [21].

The participants of this research are students from private universities that have operated in China for over three decades. With the radped development of the social economy and evolving demands of the education market, private higher universities have gained significant importance in China’s academic landscape [22]. As higher education becomes more accessible, the ‌diversification of market demands continues to rise‌, yet ‌public universities often maintain homogeneous educational models and curricula‌ that struggle to address personalized needs‌ [23]. In contrast, private institutions ‌leverage flexible structures to guarantee educational diversity‌, effectively catering to ‌students and their families‌ with varied aspirations‌. Furthermore, private higher education schools have clarified their goals, emphasized closely combining industrial needs, and strengthened the connection between education and industry to meet the employment market better [22,23]. The emergence of private higher education has dramatically enriched the education system of higher education in China, improved the quality of students’ learning, employment, and life, and to some extent, urged and promoted official efficient reforms and improvements.

This study can provides a systematic examination of the interplay between Technology Acceptance Model (TAM) and external determinants including Teacher Support Behaviors (TSBs) within China’s EDT ecosystem characterized by institutional reforms, quantifying their collective influence on online continuous learning intentions through structural equation modeling. Psychological constructs **‌**exert significant mediating effects**‌,** particularly **‌**self-motivation as a self-regulatory mechanism that facilitates cognitive engagement**‌** during digital learning processes‌ [24]. In modern society where lifelong learning competence determines developmental trajectories**‌,** students’ ability to **‌**mitigate challenges through self-regulation‌ becomes critical for sustained engagement‌ [25]. The study developed a theoretical model for continuous learning intention (CLI) in educational digital transformation (EDT) through the extension of TSBs determinants, alongside such two different factors as flow experience and TAM. It examined the influencing factors of students’ intention toward CLI within EDT and ascertained the way these variables affected students’ intention toward online continuance learning, directly or indirectly.

## 2. Literature review and hypotheses

### 2.1 Relationship between teachers’ supportive behaviors (TSBs) and perceived ease of use(PEU) and perceived usefulness(PU)

Empirical studies have consistently demonstrated that teachers are crucial in cultivating students’ academic achievement [26–28]. In both physical and virtual classrooms, ‌teachers’ supportive behaviors enhance instructional climate quality, stimulate intrinsic motivation with self-determination characteristics, and sustain self-regulated learning behaviors‌ [29]. The digital transformation of education has ‌expanded learning environments from traditional classrooms to hybrid ecosystems‌, where ‌knowledge transfer evolves into bidirectional co-construction processes‌ between teachers and learners [5,30,31]. Effective online learning stems from careful planning and promotion of teaching content and student and teacher interaction [32]. The teachers’ supportive behaviors provided in the online course environment is divided into three dimensions: teacher technical support, teacher social support, and teacher cognitive support [31,33]. Teacher technical support refers to teachers operating and managing online platforms to guide learners to use information technology tools to solve problems [34]; teacher social support relates to teachers promoting interaction and communication among students through designing activities or encouraging language [35]. Teacher cognitive support enriches and improves students’ knowledge and skills through teachers’ deep knowledge structure, personalized teaching strategies, and teaching methods to meet learners’ needs for knowledge construction and skill improvement [36]. Davis developed Davis developed the TAM in 1989 based on the model of reasoned action theory established, providing a theoretical background to explain the attitude-intention-behavior relationship [37–39]. TAM theory is used in information systems to simulate the process of individuals’ acceptance and use of information technology systems to explain and predict individuals’ attitudes and behaviors in the face of new technologies [40,41]. The central insight of the TAM is that external environmental variables in information systems affect individuals’ behavioral attitudes and decision-making intentions through perceived ease of use (PEU) and perceived usefulness (PU) [42–44].

Some researches have also indicated that supports can improve the perceived usefulness of new technologies for student groups [45,46]; teachers’ support is also an essential factor affecting the student group’s perceived ease of use of online learning technology [47,48], especially in online foreign language learning, external factors can be regarded as predictors affecting language learners’ perceptions of the convenience of using mobile phones. This study employed teachers’ supportive behaviors as external variables within TAM model, specifically examining their predictive relationships with perceived usefulness(PU) and perceived ease of use (PEU). Therefore, the following hypotheses were formulated:

**H1**: Teachers’ supportive behaviors positively affect perceived ease of use;

**H2**: Teachers’ supportive behaviors positively affect perceived usefulness.

### 2.2 Relationship between teachers’ supportive behaviors (TSBs) and flow experience (FE)

The theory of flow experience was originally proposed by the famous psychologist Sikszentmihalyih in the 1960s. He posited that when individuals channel their attention into a specific task, they enter a flow state characterized by heightened concentration, distorted temporal perception, and affective satisfaction, collectively constituting the flow experience [49–51]. Teachers’ participation in online teaching and support will enhance students’ learning efficacy by fostering confidence and refining cognitive processing [31,52]. Teachers can create a positive atmosphere through emotional support, such as encouragement, esteem-building, and personalized attention. A learning atmosphere can effectively alleviate online learning burnout [53,54]. This teachers’ support remains equally critical for cultivating metacognitive awareness necessary for academic success in virtual environments [55]. Teachers provide continuous feedback and supportive behaviors to learning groups based on learning contents and tasks, which helps to support the collaborative construction of knowledge among online learners’ groups [56]. Furthermore, the provision of diverse experiential learning opportunities not only alleviates psychosocial challenges inherent in digital education but also sustains motivational engagement [57]. In online learning, teachers’ support helps students relieve study fatigue, enhance their awareness of successful learning, and build a knowledge system to improve their attention and learning pleasure. With regard to the relationship between teachers’ support and students’ flow experience, therefore, the following hypothesis was formulated:

**H3**: Teachers’ supportive behaviors positively affect students’ flow experience.

### 2.3 Relationship between perceived ease of use(PEU), perceived usefulness (PU), and flow experience (FE)

In the TAM model, perceived usefulness refers to the extent to which an individual believes that using a particular technology will enhance their professional performance, while perceived ease of use denotes the degree to which they anticipate the system requiring minimal effort to operate effectively [58]. Empirical evidence demonstrates that perceived ease of use significantly predicts perceived usefulness in online learning ecosystems and e-learning framewords [59,60]. For instance, studies with Thai pre-service teachers reveal that the perceived usefulness of educational technology is significantly influenced by its perceived ease of use [61]. Similarly, research examining Chinese students’ adoption of Internet-based learning indicates that perceived ease of use has a substantial impact on both usefulness perceptions and behavioral attitudes [62]. Perceived ease of use was also found to significantly influence Spanish pre-service teachers’ perceptions of the usefulness of mobile technology [63]. The flow experience, rooted in an individual’s intrinsic evaluation of an activity’s goals and processes, stimulates intrinsic motivation to sustain engagement [49]. Clear goals, immediate feedback, and a balance between user skills and challenges are important triggers of flow experience [64,65]. Suppose teachers can provide timely feedback and support to students during their online learning process so that students can perceive the ease of use and usefulness of an online learning platform. In that case, it may bring them a higher flow experience. Therefore, the study formulated the following hypotheses:

**H4**: Perceived ease of use positively affects perceived usefulness;

**H5**: Perceived ease of use positively affects flow experience;

**H6**: Perceived usefulness positively affects flow experience.

### 2.4 Relationship between perceived ease of use(PEU), perceived usefulness(PU), flow experience(FE), and continuous learning intentions(CLI)

Continuous learning intention is students’ persistent commitment to engage in learning efforts for task completion [66]. Empirical studies demonstrate that CLI serves as a critical predictor for students’ sustained adoption of e-learning technologies following initial adoption [67,68]. In promoting online learning platforms, CLI not only reflects learning persistence but also indicates platform loyalty [69,70]. Research based on the TAM model confirms that attitudes affected by perceived usefulness and perceived ease of use will affect students’ online learning intentions [3,71]. As a validated theoretical framework, the TAM model is widely used in the education industry to predict student learning behaviors and attitudes in online environments [39,72].

Flow experience can effectively reduce students’ isolation and separation in virtual network environments, fostering sustained engagement in certain activities [51]. The flow experience will make students curious, happy, and focused during the online course learning process and achieve a higher learning state [49]. In educational research, flow experiences are more likely to stimulate personal motivation and achieve positive results, thereby nurturing students’ continuous learning dispositions [68,73,74]. Therefore, the following hypotheses were formulated:

**H7**: Perceived ease of use positively affects continuous learning intention;

**H8**: Perceived usefulness positively affects continuous learning intention;

**H9**: Flow experience positively affects continuous learning intention.

**Fig 1:**
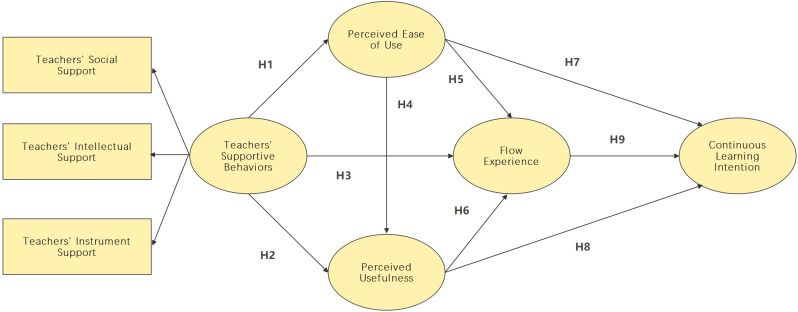
Research model.

The study explored the relationship between teachers’ supportive behaviors, perceived usefulness, perceived ease of use, perceived usefulness, flow experience, and continuous learning intention (see Fig 1).

## 3. Research methodology

### 3.1 Participants and procedures

In this study, data were collected by menas of an online questionnaire because the advantages of the online questionnaire approach are that rich data can be colleted in a shorter period of time, and data entry and processing are more convenient and accurate. This study has received approval on human research protection from the Human Research Ethics Committee, School of Foreign Languages and International Education in Hunan International Economics University, which fully complies with the principles expressed in the Declaration of Helsinki. The research targeted students pursuing bachelor’s degrees at five private universities in China. The study’s objectives of the online questionnaire and the link to the questionnaire were mostly distributed to the questioned students via their online social channels, such as WeChat, QQ, or personal email. Prior to the students completing the questionnaires, their consent as participants was informed and obtained in written form. The investigation was conducted from February 26, 2024, to March 12, 2024. After eliminating 260 unqualified or invalid questionnaires using SPSS software, a total of 614 valid questionnaires were obtained, meeting the acceptable sample size criteria.

The data collection took place from February 26, 2024, to March 12, 2024. During the data curation process, a total of 260 unqualified or invalid questionnaires were removed. The criteria used for exclusion included responses that were incomplete, inconsistent, or those that showed a lack of variability in answers (e.g., respondents who selected the same answer across all items, indicating a lack of engagement or attention). Additionally, questionnaires from participants who completed the survey in an unusually short amount of time were excluded, as these responses were deemed unreliable. To further ensure data quality, respondents with a response variance below 0.2 across all items were also removed, as such low variability suggests a lack of attention or meaningful engagement. After applying these criteria, 614 valid questionnaires remained, meeting the acceptable sample size criteria for statistical analysis.

Therefore, the questionnaire demonstrated sufficient content validity. The study used a seven-point Likert scale to measure the values of these constructs, ranging from 1 = strongly disagree to 7 = strongly agree. The Questionnaire Star website is the largest Chinese online questionnaire research platform. It can distribute questionnaires in the form of questionnaire links or questionnaire QR codes and can quickly contact the respondents through these two methods. It is often nearly impossible for researchers to determine the composition of a primary population. Since a complete catalog of students who use assisted learning platforms to learn English does not exist, where it is difficult to identify the overall sample, the study adopted a convenience sampling method for investigation. The survey subjects were college students who use auxiliary learning platforms to learn English. During the period, a total of 874 questionnaires were collected. After eliminating invalid questionnaires, answering questionnaires with duplicate IP addresses, and questionnaires that took too short a time to respond, the study obtained 614 valid questionnaires. The samples collected in the survey met sample size standards [75]. At the same time, large samples also significantly improve the power and robustness of statistical analysis [44].

### 3.2 Variable measurement

Table 1 presents the questionnaire items in the current study. This study processed the data using the partial least squares method (PLS-SEM). PLS-SEM is an iterative estimation method combining principal component analysis and multiple regression [44]. PLS has relatively lax requirements for normality and randomness of variables and also can analyze complex prediction models [76–80]. It is the primary reason why this study chose PLS-SEM as a critical data analysis tool. In the present study, PLS-SEM analysis and estimation were conducted in two stages. In the first stage, the reliability and validity of the questionnaire were analyzed. In the other stage, the structural model’s path coefficient and explanatory power were calculated and verified. The main purpose of the two stages is to demonstrate structural relationships [81,82]. This study explores the relationships among teachers’ supportive behaviors, perceived ease of use, perceived usefulness, flow experience, and continuous learning intention. Since there are many measurement indexes between each dimension and path, PLS is more suitable to reveal the relationship between relevant variables and reduce measurement errors than other SEM methods.

**Table 1:** Questionnaire items and references.

Dimensions		Questions	References
**Teachers’ Social Support**	TSS1	The teacher can guide me to study alone.	[83]
	TSS2	The teacher can help me complete my study tasks.	
	TSS3	The teacher can give me study encouragement.	
	TSS4	The teacher can praise me when I make progress in my studies.	
	TSS5	I have a good relationship with my teacher.	
**Teachers’ Intellectual Support**	TITS1	When I encounter difficult knowledge, the teacher will give me support.	
	TITS2	When I encounter learning difficulties, the teacher will support me.	
	TITS3	When I encounter learning problems, the teacher will give me support.	
	TITS4	The teacher will provide information to help me solve my learning problems.	
	TITS5	The teacher will provide me with suggestions for studying.	
**Teachers’ Instrument Support**	TISS1	When I have problems accessing the platform, the teacher will help me.	
	TISS2	When I have problems using the platform, the teacher will help me.	
	TISS3	When I encounter problems using the platform to study, the teacher will help me.	
	TISS4	The teacher will answer my questions about using the platform.	
	TISS5	The teacher will help me solve problems related to using the platform.	
**Perceived Ease of Use**	PEU1	I can use the platform easily.	[84]
	PEU2	I can use the platform flexibly.	
	PEU3	I didn’t feel any difficulty using the platform.	
	PEU4	It is straightforward for me to use the platform.	
	PEU5	It is very easy for me to use the platform.	
**Perceived Usefulness**	PU1	The platform can make my learning more efficient.	[85]
	PU2	The platform can improve my academic performance.	
	PU3	The platform can improve my learning ability.	
	PU4	It is helpful for me to use the platform to study.	
	PU5	Using the platform to learn can help me make progress.	
**Flow Experience**	FE1	When using the platform to study, we often need to remember the passage of time.	[86]
	FE2	I often don’t feel my surroundings when using a platform to study.	
	FE3	When using the platform to study, I often need to remember about other things that need to be done.	
	FE4	I concentrate on studying on the platform.	
	FE5	While studying on the platform, I didn’t think about anything else.	
**Continuous Learning Intention**	CLI1	I plan to continue to use the platform to learn in the future.	[87]
	CLI2	I am willing to continue to use the platform to learn in the future.	
	CLI3	I will often use the platform to study in the future.	
	CLI4	I am willing to continue to complete learning tasks on the platform.	
	CLI5	I can continue learning on the platform.	

## 4. Research results

### 4.1 Descriptive statistical analysis

Table 2 displays the demographic information of the students who participated in the survey. Of these students, there were 165 males, accounting for 26.9%, and 449 females, accounting for 73.1%; the monthly disposable income of most people was less than 2,000 (517, accounting for 84.2%). The most significant number of people registered in rural areas is 289, accounting for 47.1%, followed by 277 people in urban areas, accounting for 45.1%. Regarding English proficiency, 65.3% of people still need to pass proficiency in English.

**Table 2:** Participants’ demographics.

Measure	Items	Number	Percentage
**Gender**	Male	165	26.9
	Female	449	73.1
**Monthly Disposable Income**	<¥1000	140	22.8
	¥1001-¥2000	377	61.4
	¥2001-¥3000	72	11.7
	¥3001-¥5000	12	2.0
	¥5001-¥8000	2	0.3
	>¥8001	11	1.8
**Location**	City	277	45.1
	Village	289	47.1
	Suburbs	48	7.8
**English Proficiency**	CET-3	58	9.4
	CET-4	140	22.8
	CET-6	2	0.3
	TEM-8	3	0.5
	Others	10	1.6
	None	401	65.3
Total		614	100.0

### 4.2 Reliability analysis of scale data

This study analyzes five contructs: teacher supportive behaviors, perceived ease of use, perceived usefulness, flow experience, and continuous learning intention. Teacher supportive behaviors consist of teacher social support, teacher intellectual support, and teacher instrumental support [88]. According to the requirements of reliability and concurrent validity standards, standardized factor loadings exceeding 0.6 are basically acceptable and ideally should exceed 0.7 [89,90], the composite reliability should be higher than 0.6, and the average variance extracted should be higher than 0.5. When the average variance extracted > 0.5, the measurement model has excellent convergent validity [91]. The results of Table 3 indicated that the factor loadings are between 0.896–0.981, Cronbach’s α is between 0.965–0.989, Composite Reliability is between 0.973–0.991, and AVE is between 0.878–0.957, all of which meet the recommended thresholds and confirm the research questionnaire Has good reliability and convergent validity.

**Table 3:** Reliability and validity of results.

Constructs	Items	Factor Loading	Cronbach’s Alpha	rho_A	Composite Reliability	AVE
**TSS**	TSS1	0.914	0.988	0.988	0.991	0.955
	TSS2	0.954				
	TSS3	0.938				
	TSS4	0.949				
	TSS5	0.931				
**TITS**	TITS 1	0.970	0.983	0.983	0.986	0.935
	TITS 2	0.966				
	TITS 3	0.972				
	TITS 4	0.963				
	TITS 5	0.963				
**TISS**	TISS1	0.973	0.965	0.966	0.973	0.878
	TISS2	0.976				
	TISS5	0.979				
**PEU**	PEU1	0.953	0.968	0.970	0.975	0.888
	PEU2	0.955				
	PEU3	0.896				
	PEU4	0.958				
	PEU5	0.948				
**PU**	PU1	0.962	0.985	0.985	0.988	0.945
	PU3	0.979				
	PU4	0.978				
	PU5	0.977				
**FE**	FE1	0.952	0.974	0.975	0.979	0.905
	FE2	0.960				
	FE3	0.951				
	FE4	0.942				
	FE5	0.950				
**CLI**	CLI 2	0.981	0.989	0.989	0.991	0.957
	CLI 3	0.973				
	CLI 4	0.979				
	CLI 5	0.978				

Notes: TSS = teacher’s social support; TITS = teachers’ intellectual support; TISS = teachers’ instrument support; TSBs = teachers’ support behaviors; PEU = perceived ease of use; PU = perceived usefulness; FE = flow experience; CLI = continuous learning intention.

### 4.3 Discriminant reliability analysis

The present study adopted the Fornell-Larcker criterion method to measure the discriminant validity of the constructs within measurement model. When the square root of the AVE of each construct is larger than the correlation coefficient of each construct, it means that the model obtains great discriminant validity [90], as shown in Tables 4 and 5.

**Table 4:** Forell-larcker criterion.

	FE	PEU	PU	TSS	CLI
FE	0.951				
PEU	0.691	0.942			
PU	0.734	0.838	0.972		
TSB	0.724	0.763	0.842	0.904	
CLI	0.783	0.794	0.890	0.799	0.978

**Table 5:** Heterotrait-Monotrait ratio (HTMT).

	CLI	FE	PEU	PU	TSB
CLI					
FE	0.797				
PEU	0.811	0.71			
PU	0.802	0.748	0.857		
TSB	0.810	0.738	0.780	0.855	

Notes: (1) The bold values on the diagonal are the square roots of the AVE of each construct, and the others are the correlation coefficients of the constructs. (2) FE = flow experience; PEU = perceived ease of use; PU = perceived usefulness; TSS = Teachers’ Social Support; TSBs = teachers’ support behaviors; CLI = continuous learning intention.

In this study, the discriminant validity of the constructs within the measurement model was assessed using several methods, including the Fornell-Larcker criterion and the Heterotrait-Monotrait Ratio (HTMT) approach. The Fornell-Larcker criterion is a commonly used method for evaluating discriminant validity by comparing the square root of the Average Variance Extracted (AVE) of each construct to the correlations between constructs. However, it is important to note that while the Fornell-Larcker criterion is widely employed, it is not always sensitive enough to detect potential discriminant validity issues. For example, when constructs have high factor loadings, the AVE values may be inflated, potentially giving rise to false-positive results indicating good discriminant validity, even when indicators from different constructs overlap. In this study, we also considered the HTMT ratio, which is generally regarded as a more reliable method for assessing discriminant validity compared to the Fornell-Larcker criterion. The HTMT ratio evaluates the degree of correlation between constructs, and values exceeding the threshold of 0.90 indicate a lack of discriminant validity. In the current analysis, the HTMT test was conducted and its results are presented in Table 4. The findings revealed that several constructs showed HTMT values close to or exceeding the 0.90 threshold, which suggested that some constructs may not be sufficiently distinct from one another [92].

This study used the cross-loading method to measure the discriminant validity of the model. A comparison of each indicator’s cross-loadings and factor loadings showed that each scale item has a higher factor loading for its specified latent construct than for any other. When considering the loadings of the construct, this indicator has reasonable discriminant validity [91].

Specifically, indicators TISS3 and TISS4 demonstrated high cross-loadings (0.871 and 0.850, respectively) with the Teachers’ Intellectual Support (TITS) factor. This suggests that the factors “Teachers’Instrumental Support” and “Teachers’ Intellectual Support” are difficult to distinguish from one another. To address this concern and improve the model’s stability, we decided to remove TISS3, TISS4, and PU2, as these items showed problematic cross-loadings and could have compromised the model’s ability to clearly differentiate between constructs. Furthermore, the indicator CLI1 exhibited a high factor loading (0.866) with the Continuous Learning Intention (CLI) factor, which also raised concerns about discriminant validity. To mitigate these issues and ensure a stable model, CLI1 was excluded from the analysis. As shown in Table 6:

**Table 6:** Standardized factor loadings and cross loadings of the outer model.

	FE	PEU	PU	TISS	TITS	TSS	CLI
FE1	**0.952**	0.619	0.681	0.576	0.597	0.713	0.727
FE2	**0.960**	0.644	0.696	0.602	0.616	0.733	0.740
FE3	**0.951**	0.620	0.654	0.565	0.580	0.690	0.703
FE4	**0.942**	0.718	0.740	0.633	0.651	0.759	0.783
FE5	**0.950**	0.677	0.714	0.617	0.630	0.755	0.765
PEU1	0.658	**0.953**	0.807	0.662	0.738	0.704	0.772
PEU2	0.671	**0.955**	0.823	0.657	0.747	0.713	0.787
PEU3	0.627	**0.896**	0.716	0.558	0.628	0.631	0.673
PEU4	0.637	**0.958**	0.792	0.636	0.713	0.693	0.753
PEU5	0.661	**0.948**	0.804	0.635	0.715	0.703	0.752
PU1	0.719	0.814	**0.962**	0.723	0.810	0.795	0.853
PU2	0.718	0.799	**0.963**	0.723	0.781	0.807	0.866
PU3	0.712	0.806	**0.979**	0.713	0.787	0.789	0.865
PU4	0.707	0.829	**0.978**	0.734	0.808	0.790	0.874
PU5	0.711	0.824	**0.977**	0.717	0.790	0.787	0.867
TIS1	0.594	0.638	0.711	**0.973**	0.789	0.753	0.652
TIS2	0.600	0.633	0.708	**0.976**	0.800	0.760	0.655
TIS3	0.621	0.657	0.740	**0.981**	0.823	0.783	0.682
TIS4	0.629	0.672	0.734	**0.977**	0.826	0.796	0.686
TIS5	0.635	0.670	0.737	**0.979**	0.824	0.795	0.688
TITS1	0.634	0.725	0.785	0.827	**0.970**	0.845	0.744
TITS2	0.624	0.717	0.771	0.806	**0.966**	0.830	0.734
TITS3	0.636	0.735	0.795	0.814	**0.972**	0.847	0.752
TITS4	0.621	0.728	0.804	0.796	**0.963**	0.822	0.748
TITS5	0.617	0.734	0.803	0.777	**0.963**	0.814	0.758
TISS1	0.720	0.643	0.714	0.721	0.730	**0.914**	0.694
TISS2	0.734	0.678	0.759	0.759	0.792	**0.954**	0.742
TISS3	0.698	0.706	0.793	0.743	0.871	**0.938**	0.774
TISS4	0.711	0.709	0.797	0.779	0.850	**0.949**	0.762
TISS5	0.740	0.690	0.759	0.727	0.780	**0.931**	0.759
CLI1	0.759	0.779	0.870	0.684	0.760	0.773	**0.978**
CLI2	0.754	0.778	0.873	0.675	0.761	0.778	**0.981**
CLI3	0.772	0.777	0.870	0.681	0.763	0.791	**0.973**
CLI4	0.780	0.778	0.870	0.662	0.750	0.776	**0.979**
CLI5	0.764	0.773	0.870	0.665	0.745	0.779	**0.978**

Notes:

(1)The bold cells are the factor loadings of the scale items for each construct. (2) FE = flow experience; PEU = perceived ease of use; PU = perceived usefulness; TISS = teachers’ instrument support; TSS = teachers’ social support; CLI = continuous learning intention.

### 4.4 Path analysis

To evaluate the model structure, the study used the bootstrapping procedure of 5000 repeated samples to obtain the standard β (β), t value, and coefficient of determination (R2) [93]. Meanwhile the study used the model to estimate path coefficients and T values. The path coefficient represents the strength and direction of the relationship between variables to show the relationship between observed variables and latent variables. The R2 value is the percentage that explains the dependent variable, representing the predictive ability of the model.

**Fig 2:**
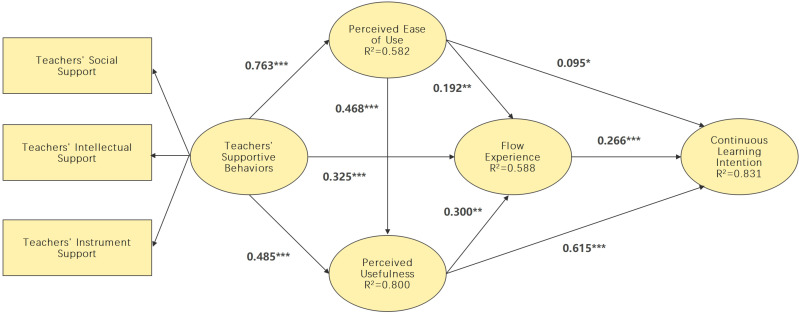
Standardized path coefficients and significance.

It can be seen from Tables 6 and 7, and Fig 2 that teachers’ support behaviors positively affect perceived ease of use, supporting H1 (TSBs → PEU: β = 0.763, t value = 27.312). Teachers’ support behaviors significantly impact perceived usefulness H2 (TSBs → PU: β = 0.488, t value = 8.957). Teachers’ support behaviors especially impact flow experience, supporting H3 (TSBs → FE: β = 0.325, t value = 4.300). Perceived ease of use significantly impacts perceived ease of use, helping H4 (PU → PEU: β = 0.468, t value = 8.246). Perceived ease of use substantially affects flow experience, supporting H5 (PEU → FE: β = 0.192, t value = 2.827). Perceived usefulness significantly impacts flow experience, supporting H6 (PU → FE: β = 0.300, t value = 3.280). Perceived ease of use significantly impacts continuous learning intention, supporting H7 (PEU → CLI: β = 0.095, t value = 2.011). Perceived usefulness significantly impacts continue learning intention, supporting H8 (PU → CLI: β = 0.615, t value = 11.659). Flow experience significantly affects continuous learning intention, supporting H9 (FE → CLI: β = 0.266, t value = 6.119).

**Table 7:** Model hypotheses testing results.

Hypothesis	Relationship	Standardized Path Coefficient	Sample Mean (M)	Standard Deviation (STDEV)	T Statistics	P Values	Decision
**H1**	TSBs - > PEU	0.763	0.763	0.028	27.312***	0.000	Supported
**H2**	TSBs - > PU	0.485	0.488	0.054	8.957***	0.000	Supported
**H3**	TSBs - > FE	0.325	0.328	0.076	4.300***	0.000	Supported
**H4**	PU - > PEU	0.468	0.465	0.057	8.246***	0.000	Supported
**H5**	PEU - > FE	0.192	0.193	0.068	2.827**	0.005	Supported
**H6**	PU - > FE	0.300	0.295	0.091	3.280**	0.001	Supported
**H7**	PEU - > CLI	0.095	0.095	0.047	2.011*	0.044	Supported
**H8**	PU - > CLI	0.615	0.615	0.053	11.659***	0.000	Supported
**H9**	FE - > CLI	0.266	0.267	0.043	6.119***	0.000	Supported

Notes: (1)TSBs = teachers’ support behaviors; PEU = perceived ease of use; PU = perceived usefulness; FE = flow experience; CLI = continuous learning intention. (2)*p-value < 0.05; ** p-value < 0.01; *** p-value < 0.001。

Table 8 presents the results of the mediating effect analysis. The findings indicate several significant indirect relationships. Specifically, Teachers’ Supportive Behaviors (TSBs) positively influence Perceived Usefulness (PU), which in turn affects Flow Experience (FE) with a β = 0.145, t = 3.334, and p = 0.001, supporting the mediation hypothesis. Additionally, TSBs influence Perceived Ease of Use (PEU), which also significantly impacts Flow Experience (FE), with a β = 0.147, t = 2.830, and p = 0.005. Furthermore, TSBs indirectly affect Continuous Learning Intention (CLI) through both Perceived Ease of Use (PEU) and Perceived Usefulness (PU), with β values of 0.298 (t = 6.817, p < 0.001) and 0.073 (t = 1.993, p = 0.046), respectively. Lastly, Flow Experience (FE) significantly mediates the relationship between TSBs and CLI, with β = 0.086, t = 3.322, and p = 0.001. These results confirm that TSBs have an indirect influence on students’ learning intentions, mainly through perceptions of ease of use, usefulness, and flow experience.

**Table 8:** Mediating effect.

	Original Sample (O)	Sample Mean (M)	Standard Deviation (STDEV)	T Statistics (|O/STDEV)	P Values	test result
TSBs - > PU - > FE	0.145	0.142	0.044	3.334**	0.001	Supported
TSBs - > PEU - > FE	0.147	0.147	0.052	2.830 **	0.005	Supported
TSBs - > PU - > CLI	0.298	0.300	0.044	6.817***	0.000	Supported
TSBs - > PEU - > CLI	0.073	0.072	0.037	1.993*	0.046	Supported
TSBs - > PEU - > PU	0.357	0.354	0.038	9.278***	0.000	Supported
TSBs - > FE - > CLI	0.086	0.088	0.026	3.322**	0.001	Supported
PEU - > PU - > FE	0.140	0.138	0.049	2.858**	0.004	Supported
PEU- > FE - > CLI	0.051	0.052	0.020	2.514*	0.012	Supported
PEU - > PU - > CLI	0.288	0.286	0.043	6.765***	0.000	Supported
PU - > FE - > CLI	0.080	0.079	0.027	2.929**	0.003	Supported

Notes: (1)TSBs = teachers’ support behaviors; PEU = perceived ease of use; PU = perceived usefulness; FE = flow experience; CLI = continuous learning intention. (2)*p-value < 0.05; ** p-value < 0.01; *** p-value < 0.001.

## 5. Discussion

This study investigates the factors that affect students’ intention to continue learning online under the digital transformation of education and how these variables affect students’ intention to continue learning online. Nine hypotheses were proposed and supported after data collection and empirical research analysis.

Firstly, the results show that TSBs significantly impact PEU, PU, and FE (H1, H2, H3). Therefore, the results of this study are in line with previous research in which teachers play a vital role as their support to students may influence students’ learning process [67]. The study found that teacher intellectual support had no direct impact on online continuous learning intention but can indirectly affect online continuous learning intentions through PEU, PU, and FE, which illustrates the personal knowledge literacy of teachers in online courses, teaching strategies, and methods can effectively help students master learning content. Promoting PEU can effectively enhance PU and FE, thereby improving students’ online learning persistence, which is in accord with Huang and Yao’s research conclusion [44,47]. On the one hand, teachers’ knowledge brings supplementary knowledge to students in online courses and expands the scope of students’ knowledge; on the other hand, teachers help students construct cognitive scaffolds through carefully designed teaching strategies and teaching methods, making it easier for students to achieve learning objectives, promote knowledge mastery and ability improvement. Therefore, besides possessing professional knowledge and teaching abilities, teachers must also be good at using teaching methods and strategies to stimulate students to continue learning. Meanwhile, students in a virtual learning space also expect social support. The communication, collaboration, communication, and interaction between teachers and students, students and students, are always strong links to promote students’ efficient learning. The expectation of a “spontaneous learning process expectation” after taking the course is consistent with Jiang’s research [14,94]. Enhancing teachers’ instrumental support can help improve PEU and PU, thereby further affecting the intention to continue learning, which is consistent with Conceicao’s research [52]. In addition to directly affecting PEU and PU, teacher instrumental support also indirectly affects FE through PEU, which shows that in the online learning environment supported by information technology, technology serves as the fundamental guarantee for online learning to occur, and instrumental support is the primary support behavior provided by teachers [52]. By helping students solve operational problems about information technology usage during the learning process, teachers ensure that the learning process is not affected by changes in the external technical environment, directly or indirectly promoting PEU, PU, and FE and ultimately affecting continuance learning intention [39,72]. Therefore, besides providing necessary learning management support, teachers must give technical tools for knowledge construction and management and guide students to use them actively, thus encouraging students to continue learning. TSBs can stimulate students’ continuous learning motivation, enable them to maintain interest and enthusiasm for learning, actively invest in learning, and have a specific self-motivation effect on students’ continuous learning [24].

Secondly, the results show that students’ online perceived ease of use positively correlates with perceived usefulness within educational digital transformation (H4). Moreover, PEU and PU also have a significant relationship with students’ FE in online learning (H5, H6). In addition, PEU and PU are also strong predictors of students’ intentions to continue learning online under the digital transformation of education (H7, H8). These findings are consistent with TAM and with other relevant literature reviews. Researchers have widely confirmed the correlation between PEU, PU, and behavioral intention. PEU significantly affects PU and FE, supporting Thi and Al-Emran’s research conclusion that PEU in online learning affects learners’ FE [3,71]. Therefore, PEU is the core and foundation of students’ intention to continue learning online and plays an essential part in enhancing students’ intention to continue learning in online courses by promoting PU and FE. PEU is a critical factor in learners’ intention of continuing learning. Alffada pointed out that persistence in online course learning is directly affected by PU which mediates the impact of PEU on learning persistence [37]. We can also speculate that students’ PEU is to put first online learning; it is necessary to conduct the research for extensive and in-depth demand, to design challenging, diversified content and activities suitable for students’ levels to influence students’ perception of usefulness, stimulates students’ curiosity and concentration, continuously improving the quality of online learning and promoting the continuance of learning behaviors, which will have a specific positive effect on students’ self-motivation [25].

Thirdly, research also shows that students’ FE significantly affects their intention to continue learning online (H9), which aligns with the conditions for flow experience proposed by Csikszentmihalyi, namely a positive learning atmosphere, a harmonious teacher-student relationship, and motivational leadership. Online teacher-student communication promotes flow experience by enhancing students’ positive emotional experience [86]. The research results are also consistent with Csikszentmihalyi and Finneran’s research, and teachers’ online support is the main factor affecting flow experience [51,95]. Specifically, teachers should focus on designing learning content and activities that enhance students’ emotional experience in course design, such as personalized expression of self-introduction, goal commitment, establishment of learning contracts, personalized learning reflection, and teachers who use humorous language. Viewpoints can cultivate a positive and relaxed learning atmosphere to establish strong social connections with students through live teaching or online communication tools, thus enhancing students’ enjoyment, satisfaction and sustained motivation with online learning, promoting the continuity of online learning, and positively affecting students’ self-motivation.

## 6. Conclusions and limitations

The present study elucidated the influential factors that affect students’ online continuous learning intention based on TAM under educational digital transformation. The results revealed that (a) the constructs, including TAM, specifically perceived ease of use and perceived usefulness, had a positive impact on student’s intention of online continuance learning within educational digital transformation; (b) teachers’ supportive behaviors, including teachers’ intellectual support, teacher social support, and teacher instrument support, toward online continuous learning had a positive effect on perceived ease of use, perceived usefulness, and flow experience; (c) flow experience demonstrated a positive predictive relationship with students’ intention toward online continuance learning.

The present findings further verify the pivotal role of the examined variables in influencing students’ intention toward CLI during an educational digital transformation, with particular emphasis on the salience of teacher support behaviors, perceived ease of use, and perceived usefulness. This empirical investigation yields valuable insights for academicians and policymakers, demonstrating that these factors can enhance students’ learning processes. It is recommended that educational institutions cultivate and strategically leverage these identified elements to facilitate students’ learning progress and elevate their academic performance.

The contributions of this study are as follows: first, empirical evidence elucidating the relation of teacher support behaviors, perceived ease of use, perceived usefulness, and flow experience to students’ online continious learning intention in an educational digital transformation. We found that these elements collectively and significantly influenced students’ intention toward online continuance learning. Thus, the key to equipping students with more intention of engaging in online learning in an educational digital transformation lies in such influential factors as teachers, teacher intellectual support, teacher social support, and teacher instrumental support, alongside optimizing students’ affective experiences during online learning. To maximize learning outcomes, institutions should not only strengthen positive support mechanisms but also guide students in selecting appropriate online courses or adapting to existing ones, thereby promoting active participation and academic success. Furthermore, it is also beneficial to assist students in improving their learning capabilities to feel more engaged in online continuance learning in the educational digital transformation. Secondly, this study enriches CLI literature by proposing a novel theoretical model that extends the Technology Acceptance Model (TAM). This integrated framework addresses both psychological dimensions through TAM constructs and external contextual factors through TSBs, surpassing traditional TAM applications. By incorporating critical variables with demonstrated influence on CLI within digital transformation contexts, the model offers a more comprehensive explanatory mechanism.

The present study contributes to the literature on factors influencing students’ intention toward online continuance learning based on educational digital transformation, both empirically and theoretically. However, several limitations should be acknowledged. Firstly, the sample selection in this study was constrained to 614 students from five private universities in three Chinese cities, specifically targeting bachelor’s degree candidates. This sampling approach excludes public university students, potentially limiting the generalizability of findings. It is suggested that future research should consider expanding the sample to include students from public universities, at different academic levels, as well as from across the whole country or even from other countries with varying cultures or educational systems, which allows for the quantitative data that can be used to draw a broader conclusion and yield more macro-level analyses, and thus enhance the ecological validity of the study. Secondly, while this study integrated teacher support behaviors and flow experience into the theoretical model, other critical contextual factors remain unexplored. Variables such as educational policies, institutional facilitating conditions, campus culture, and learner self-efficacy warrant inclusion in subsequent models to provide a more holistic understanding of CLI intention dynamics during digital transformation. Thirdly, qualitative approaches in this field can also be adopted to explore students’ online continuance learning intentions further. Furthermore, the findings’ generalizability should be interpreted with caution due to the sample’s specific demographic characteristics and geographic limitation to private Chinese universities.

## Supporting information

S1 Data Raw data. (CSV)

## References

[1] Goopio J, Cheung C. The MOOC dropout phenomenon and retention strategies. J Teach Travel Tour. 2020;21(2):177–97. doi: 10.1080/15313220.2020.1809050

[2] Zhao P, et al. Precise recognition model for mobile learning procrastination based on backpropagation neural network. Sens Mater. 2023;35.

[3] Al-Emran M, Arpaci I, Salloum SA. An empirical examination of continuous intention to use m-learning: An integrated model. Educ Inf Technol. 2020;25:2899–918.

[4] Prenkaj B, Velardi P, Stilo G, Distante D, Faralli S. A survey of machine learning approaches for student dropout prediction in online courses. ACM Comput Surv. 2020;53(3):1–34. doi: 10.1145/3388792

[5] Wang P, Zhao P, Li Y. Design of education information platform on education big data visualization. Wirel Commun Mob Comput. 2022;2022:1–13. doi: 10.1155/2022/6779105

[6] Guo Q, Zeng Q, Zhang L. What social factors influence learners’ continuous intention in online learning? A social presence perspective. ITP. 2022;36(3):1076–94. doi: 10.1108/itp-02-2021-0151

[7] Esra M, Sevilen Ç. Factors influencing EFL students’ motivation in online learning: a qualitative case study. J Educ Technol Online Learn. 2021;4(1):11–22.

[8] Zhu Y, Zhang JH, Au W, Yates G. University students’ online learning attitudes and continuous intention to undertake online courses: a self-regulated learning perspective. Educ Tech Res Dev. 2020;68(3):1485–519. doi: 10.1007/s11423-020-09753-w

[9] Geng S, Law KMY, Niu B. Investigating self-directed learning and technology readiness in blending learning environment. Int J Educ Technol High Educ. 2019;16(1):1–22. doi: 10.1186/s41239-019-0147-0

[10] Chen T, et al. Analysis of user satisfaction with online education platforms in China during the COVID-19 pandemic. In: Healthcare. MDPI; 2020. 10.3390/healthcare8030200 PMC7551570 32645911

[11] Wan L, Xie S, Shu A. Toward an understanding of university students’ continued intention to use MOOCs: when UTAUT model meets TTF model. Sage Open. 2020;10(3):2158244020941858.

[12] Krouska A, Troussas C, Sgouropoulou C. Mobile game-based learning as a solution in COVID-19 era: modeling the pedagogical affordance and student interactions. Educ Inf Technol. 2022:1–13. 10.1007/s10639-021-10672-3 PMC8299167 34316285

[13] Alamri MM. Investigating students’ adoption of MOOCs during COVID-19 pandemic: students’ academic self-efficacy, learning engagement, and learning persistence. Sustainability. 2022;14(2):714. doi: 10.3390/su14020714

[14] Jiang Y, Liu H, Yao Y, Li Q, Li Y. The positive effects of growth mindset on students’ intention toward self-regulated learning during the COVID-19 pandemic: a PLS-SEM approach. Sustainability. 2023;15(3):2180. doi: 10.3390/su15032180

[15] Baber H. Determinants of students’ perceived learning outcome and satisfaction in online learning during the pandemic of COVID-19. J Educ e-learn Res. 2020;7(3):285–92.

[16] Suriagiri S, et al. Online vs. in-campus, comparative analysis of intrinsic motivation inventory, student engagement and satisfaction: a way forward for post COVID-19 era. EJEL. 2022;20(5):588–604.

[17] Wang T, Lin CL, Su YS. Continuance intention of university students and online learning during the COVID-19 pandemic: a modified expectation confirmation model perspective. Sustainability. 2021;13(8):4586.

[18] Aldhahi MI, Alqahtani AS, Baattaiah BA, Al-Mohammed HI. Exploring the relationship between students’ learning satisfaction and self-efficacy during the emergency transition to remote learning amid the coronavirus pandemic: a cross-sectional study. Educ Inf Technol (Dordr). 2022;27(1):1323–40. doi: 10.1007/s10639-021-10644-7 34276239 PMC8275635

[19] Shahzad A, Hassan R, Aremu AY, Hussain A, Lodhi RN. Effects of COVID-19 in E-learning on higher education institution students: the group comparison between male and female. Qual Quant. 2021;55(3):805–26. doi: 10.1007/s11135-020-01028-z 32836471 PMC7402545

[20] Rajeh MT, Abduljabbar FH, Alqahtani SM, Waly FJ, Alnaami I, Aljurayyan A, et al. Students’ satisfaction and continued intention toward e-learning: a theory-based study. Med Educ Online. 2021;26(1):1961348. doi: 10.1080/10872981.2021.1961348 34338161 PMC8330719

[21] Ferrer J, et al. Students’ motivation and engagement in higher education: the importance of attitude to online learning. High Educ. 2020:1–22.

[22] Jiang H, et al. Online learning satisfaction in higher education during the COVID-19 pandemic: a regional comparison between Eastern and Western Chinese universities. Educ Inf Technol. 2021:1–23. 10.1007/s10639-021-10519-x PMC8010491 33814959

[23] Su Y, Zhu Z, Chen J, Jin Y, Wang T, Lin C-L, et al. Factors influencing entrepreneurial intention of university students in China: integrating the perceived university support and theory of planned behavior. Sustainability. 2021;13(8):4519. doi: 10.3390/su13084519

[24] Meece JL. The role of motivation in self-regulated learning. In: Self-regulation of learning and performance. Routledge; 2023. p. 25–44.

[25] Seli H. Motivation and learning strategies for college success: a focus on self-regulated learning. Routledge; 2019.

[26] Hattie J. Visible learning: a synthesis of over 800 meta-analyses relating to achievement. Routledge; 2008.

[27] Nye B, Konstantopoulos S, Hedges LV. How large are teacher effects? Educ Eval Policy Anal. 2004;26(3):237–57. doi: 10.3102/01623737026003237

[28] Roorda DL, Jak S, Zee M, Oort FJ, Koomen HMY. Affective teacher–student relationships and students’ engagement and achievement: a meta-analytic update and test of the mediating role of engagement. SPR. 2017;46(3):239–61. doi: 10.17105/spr-2017-0035.v46-3

[29] Fu D, Liu Y, Zhang D. The relationship between teacher autonomy support and student mathematics achievement: a 3-year longitudinal study. Educ Psychol. 2023;43(2–3):187–206. doi: 10.1080/01443410.2023.2190064

[30] Castro Benavides LM, Tamayo Arias JA, Arango Serna MD, Branch Bedoya JW, Burgos D. Digital transformation in higher education institutions: a systematic literature review. Sensors (Basel). 2020;20(11):3291. doi: 10.3390/s20113291 32526998 PMC7309098

[31] Dughi T, Rad D, Runcan R, Chiș R, Vancu G, Maier R, et al. A network analysis-driven sequential mediation analysis of students’ perceived classroom comfort and perceived faculty support on the relationship between teachers’ cognitive presence and students’ grit-a holistic learning approach. Behav Sci (Basel). 2023;13(2):147. doi: 10.3390/bs13020147 36829376 PMC9951853

[32] Swan K. Learning effectiveness online: what the research tells us. Elem Q Online Educ Pract Dir. 2003;4(1):13–47.

[33] Duckworth A, Gross JJ. Self-control and grit: related but separable determinants of success. Curr Dir Psychol Sci. 2014;23(5):319–25. doi: 10.1177/0963721414541462 26855479 PMC4737958

[34] Myers D, Sugai G, Simonsen B, Freeman J. Assessing teachers’ behavior support skills. TESE. 2017;40(2):128–39. doi: 10.1177/0888406417700964

[35] Qureshi MA, Khaskheli A, Qureshi JA, Raza SA, Yousufi SQ. Factors affecting students’ learning performance through collaborative learning and engagement. Interact Learn Environ. 2021;31(4):2371–91. doi: 10.1080/10494820.2021.1884886

[36] Hettinger K, Lazarides R, Schiefele U. Motivational climate in mathematics classrooms: teacher self-efficacy for student engagement, student- and teacher-reported emotional support and student interest. ZDM Math Educ. 2022;55(2):413–26. doi: 10.1007/s11858-022-01430-x

[37] Levy R, Asman O, Barnoy S. Boundary-blurred behaviors in academic teachers-students facebook interaction: are guidelines needed? A cross-sectional study. BMC Nurs. 2024;23(1):816. doi: 10.1186/s12912-024-02466-y 39516774 PMC11549866

[38] Davis FD, Bagozzi RP, Warshaw PR. User acceptance of computer technology: a comparison of two theoretical models. Manag Sci. 1989;35(8):982–1003. doi: 10.1287/mnsc.35.8.982

[39] Alfadda HA, Mahdi HS. Measuring students’ use of zoom application in language course based on the Technology Acceptance Model (TAM). J Psycholinguist Res. 2021;50(4):883–900. doi: 10.1007/s10936-020-09752-1 33398606 PMC7781650

[40] Granić A. Educational technology adoption: a systematic review. Educ Inf Technol (Dordr). 2022;27(7):9725–44. doi: 10.1007/s10639-022-10951-7 35399780 PMC8979725

[41] Marangunić N, Granić A. Technology acceptance model: a literature review from 1986 to 2013. Univ Access Inf Soc. 2015;14:81–95.

[42] Ren L, Yang F, Gu C, Sun J, Liu Y. A study of factors influencing Chinese college students’ intention of using metaverse technology for basketball learning: extending the technology acceptance model. Front Psychol. 2022;13:1049972. doi: 10.3389/fpsyg.2022.1049972 36605282 PMC9808391

[43] Nikou S, De Reuver M, Mahboob Kanafi M. Workplace literacy skills—how information and digital literacy affect adoption of digital technology. JD. 2022;78(7):371–91. doi: 10.1108/jd-12-2021-0241

[44] Yao Y, Wang P, Jiang Y, Li Q, Li Y. Innovative online learning strategies for the successful construction of student self-awareness during the COVID-19 pandemic: merging TAM with TPB. J Innov Knowl. 2022;7(4):100252. doi: 10.1016/j.jik.2022.100252

[45] Almulla MA. Investigating important elements that affect students’ readiness for and practical use of teaching methods in higher education. Sustainability. 2022;15(1):653. doi: 10.3390/su15010653

[46] Keržič D, Tomaževič N, Aristovnik A, Umek L. Exploring critical factors of the perceived usefulness of blended learning for higher education students. PLoS One. 2019;14(11):e0223767. doi: 10.1371/journal.pone.0223767 31751345 PMC6872162

[47] Huang F, Teo T, Scherer R. Investigating the antecedents of university students’ perceived ease of using the Internet for learning. Interact Learn Environ. 2022;30(6):1060–76.

[48] Tarhini A, Hone K, Liu X. A cross‐cultural examination of the impact of social, organisational and individual factors on educational technology acceptance between British and Lebanese university students. Br J Educ Technol. 2014;46(4):739–55. doi: 10.1111/bjet.12169

[49] Management, A.O. The academy of management review, vol. 8. Academy of Management; 1983.

[50] Kim D, Ko YJ. The impact of virtual reality (VR) technology on sport spectators’ flow experience and satisfaction. Comput Human Behav. 2019;93:346–56. doi: 10.1016/j.chb.2018.12.040

[51] Csikszentmihalyi M. Applications of flow in human development and education. Springer; 2014.

[52] Conceição SC, Howles L. Designing the online learning experience: evidence-based principles and strategies. Taylor & Francis; 2023.

[53] Hung CY, et al. Compacting, picking and growing for unforgetting continual learning. Adv Neural Inf Process Syst. 2019;32.

[54] Yang G, Sun W, Jiang R. Interrelationship amongst university student perceived learning burnout, academic self-efficacy, and teacher emotional support in china’s english online learning context. Front Psychol. 2022;13:829193. doi: 10.3389/fpsyg.2022.829193 35360629 PMC8963801

[55] Armah JK, Bervell B, Bonsu NO. Modelling the role of learner presence within the community of inquiry framework to determine online course satisfaction in distance education. Heliyon. 2023;9(5):e15803. doi: 10.1016/j.heliyon.2023.e15803 37180887 PMC10172789

[56] Hernández-Sellés N, Muñoz-Carril PC, González-Sanmamed M. Computer-supported collaborative learning: an analysis of the relationship between interaction, emotional support and online collaborative tools. Comput Educ. 2019;138:1–12. doi: 10.1016/j.compedu.2019.04.012

[57] Özhan ŞÇ, Kocadere SA. The effects of flow, emotional engagement, and motivation on success in a gamified online learning environment. J Educ Comput Res. 2019;57(8):2006–31. doi: 10.1177/0735633118823159

[58] Akther T, Nur T. A model of factors influencing COVID-19 vaccine acceptance: a synthesis of the theory of reasoned action, conspiracy theory belief, awareness, perceived usefulness, and perceived ease of use. PLoS One. 2022;17(1):e0261869. doi: 10.1371/journal.pone.0261869 35020764 PMC8754289

[59] Jiang MY-C, Jong MS-Y, Lau WW-F, Meng Y-L, Chai C-S, Chen M. Validating the general extended technology acceptance model for E-learning: evidence from an online English as a Foreign language course amid COVID-19. Front Psychol. 2021;12:671615. doi: 10.3389/fpsyg.2021.671615 34658995 PMC8517242

[60] Yim JSC, Moses P, Azalea A. Predicting teachers’ continuance in a virtual learning environment with psychological ownership and the TAM: a perspective from Malaysia. Educ Technol Res Dev. 2019;67(3):691–709.

[61] Khlaisang J, Teo T, Huang F. Acceptance of a flipped smart application for learning: a study among Thai university students. Interact Learn Environ. 2021;29(5):772–89.

[62] Huang F, Teo T, Zhou M. Chinese students’ intentions to use the internet-based technology for learning. Educ Tech Res Dev. 2019;68(1):575–91. doi: 10.1007/s11423-019-09695-y

[63] Sánchez‐Prieto JC, Huang F, Olmos‐Migueláñez S, García‐Peñalvo FJ, Teo T. Exploring the unknown: the effect of resistance to change and attachment on mobile adoption among secondary pre‐service teachers. Br J Educ Tech. 2019;50(5):2433–49. doi: 10.1111/bjet.12822

[64] Autin KL, Herdt ME, Garcia RG, Ezema GN. Basic psychological need satisfaction, autonomous motivation, and meaningful work: a self-determination theory perspective. J Career Assess. 2021;30(1):78–93. doi: 10.1177/10690727211018647

[65] Aubrey S. Dynamic engagement in second language computer-mediated collaborative writing tasks: Does communication mode matter? Stud Second Lang Learn Teach. 2022;12(1):59–86. doi: 10.14746/ssllt.2022.12.1.4

[66] Hwang G-J, Wang S-Y, Lai C-L. Effects of a social regulation-based online learning framework on students’ learning achievements and behaviors in mathematics. Comput Educ. 2021;160:104031. doi: 10.1016/j.compedu.2020.104031

[67] Jiang Y, Wang P, Li Q, Li Y. Students’ intention toward self-regulated learning under blended learning setting: PLS-SEM approach. Sustainability. 2022;14(16):10140. doi: 10.3390/su141610140

[68] Junn H. L2 communicative competence analysis via synchronous computer-mediated communication (SCMC) as an alternative to formal classrooms. Innov Lang Learn Teach. 2021;17(1):15–31. doi: 10.1080/17501229.2021.1895802

[69] Wang X, et al. Perceived usefulness predicts second language learners’ continuance intention toward language learning applications: a serial multiple mediation model of integrative motivation and flow. Educ Inf Technol. 2022:1–17.

[70] Zong Y, Xing H. Customer stratification theory and value evaluation—analysis based on improved RFM model. IFS. 2021;40(3):4155–67. doi: 10.3233/jifs-200737

[71] Thi HP, et al. Factors motivating students’ intention to accept online learning in emerging countries: the case study of Vietnam. J Appl Res High Educ. 2023;15(2):324–41.

[72] Turner M, Kitchenham B, Brereton P, Charters S, Budgen D. Does the technology acceptance model predict actual use? A systematic literature review. Inf Softw Technol. 2010;52(5):463–79. doi: 10.1016/j.infsof.2009.11.005

[73] Kong S, Wang Y. The influence of parental support and perceived usefulness on students’ learning motivation and flow experience in visual programming: investigation from a parent perspective. Br J Educ Technol. 2021;52(4):1749–70. doi: 10.1111/bjet.13071

[74] Ha Y, Im H. The role of an interactive visual learning tool and its personalizability in online learning: flow experience. OLJ. 2020;24(1):205–26. doi: 10.24059/olj.v24i1.1620

[75] Krejcie RV, Morgan DW. Determining sample size for research activities. Educ Psychol Meas. 1970;30(3):607–10.

[76] Gefen D, Rigdon EE, Straub D. Editor’s comments: an update and extension to SEM guidelines for administrative and social science research. MIS Q. 2011;35(2):iii. doi: 10.2307/23044042

[77] Hair JF, Risher JJ, Sarstedt M, Ringle CM. When to use and how to report the results of PLS-SEM. EBR. 2019;31(1):2–24. doi: 10.1108/ebr-11-2018-0203

[78] Chin WW, Newsted PR. Structural equation modeling analysis with small samples using partial least squares. Stat Strat Small Samp Res. 1999;1(1):307–41.

[79] Petter S, Straub D, Rai A. Specifying formative constructs in information systems research. MIS Q. 2007:623–56.

[80] Khan GF, Sarstedt M, Shiau W-L, Hair JF, Ringle CM, Fritze MP. Methodological research on partial least squares structural equation modeling (PLS-SEM). Internet Res. 2019;29(3):407–29. doi: 10.1108/intr-12-2017-0509

[81] Anderson JC, Gerbing DW. Structural equation modeling in practice: a review and recommended two-step approach. Psychol Bull. 1988;103(3):411–23. doi: 10.1037/0033-2909.103.3.411

[82] Hulland J. Use of partial least squares (PLS) in strategic management research: a review of four recent studies. Strat Mgmt J. 1999;20(2):195–204. doi: 10.1002/(sici)1097-0266(199902)20:2<195::aid-smj13>3.0.co;2-7

[83] Sugai G, Horner RH. Defining and describing schoolwide positive behavior support. In: Handbook of positive behavior support. Springer; 2009. p. 307–26.

[84] Larcker DF, Lessig VP. Perceived usefulness of information: a psychometric examination*. Decis Sci. 1980;11(1):121–34. doi: 10.1111/j.1540-5915.1980.tb01130.x

[85] Venkatesh V, Davis FD. A model of the antecedents of perceived ease of use: development and test. Decis Sci. 1996;27(3):451–81. doi: 10.1111/j.1540-5915.1996.tb01822.x

[86] Csikszentmihalyi M. The flow experience and its significance for human psychology. In: Optimal experience: Psychological studies of flow in consciousness, vol. 2; 1988. p. 15–35.

[87] Ngah AH, Kamalrulzaman NI, Mohamad MFH, Rashid RA, Harun NO, Ariffin NA, et al. The sequential mediation model of students’ willingness to continue online learning during the COVID-19 pandemic. Res Pract Technol Enhanc Learn. 2022;17(1):13. doi: 10.1186/s41039-022-00188-w 35350391 PMC8947953

[88] Li H. Research on the influence of consumer confusion on consumer negative word-of-mouth dissemination--the mediating role of perceived gain and loss. 2023.

[89] Chin WW. The partial least squares approach to structural equation modeling. Mod Methods Bus Res. 1998;295(2):295–336.

[90] Fornell C, Larcker DF. Evaluating structural equation models with unobservable variables and measurement error. J Mark Res. 1981;18(1):39–50. doi: 10.1177/002224378101800104

[91] Hair JF, et al. Exploratory data analysis in business and economics. In: Multivariate data analysis. Pearson; 2014. p. 23–60.

[92] Gold AH, Malhotra A, Segars AH. Knowledge management: An organizational capabilities perspective. J Manag Inf Syst. 2001;18(1):185–214.

[93] Hair J, Hollingsworth CL, Randolph AB, Chong AYL. An updated and expanded assessment of PLS-SEM in information systems research. IMDS. 2017;117(3):442–58. doi: 10.1108/imds-04-2016-0130

[94] Ali MM, Mahmood MA, Anwar MN, Khan LA, Hussain A. Pakistani learners’ perceptions regarding mobile assisted language learning in ESL classroom. IJEL. 2019;9(4):386. doi: 10.5539/ijel.v9n4p386

[95] Csíkszentmihályi M. Flow: psicologia dell’esperienza ottimale. Roi edizioni; 2022.

